# The complete chloroplast genome of *Reevesia thyrsoidea* (Malvaceae)

**DOI:** 10.1080/23802359.2019.1674206

**Published:** 2019-12-13

**Authors:** Guoming Quan, Peishan Zou, Guofeng Liu, Miaomiao Sun, Wei Wang, Seping Dai

**Affiliations:** a Department of Urban Construction Engineering, Guangzhou City Polytechnic, Guangzhou, Guangdong, China;; b Guangzhou Institute of Forestry and Landscape Architecture, Guangzhou, Guangdong, China

**Keywords:** *Reevesia thyrsoidea*, ornamental plant, complete chloroplast genome, automated assembly

## Abstract

*Reevesia thyrsoidea* Lindl. is an important ornamental plant with horticultural, industrial, and timber usages. In this study, we reported a complete chloroplast genome of *R. thyrsoidea*, which was quadripartite and 161,786 bp in size, including two inverted repeats (25,466 bp for each) that separated one large single-copy (90,565 bp) and one small single-copy (20,289 bp) regions. The chloroplast genome contained 131 unique genes (86 protein-coding, 37 tRNA, and 8 rRNA), and 17 of them were double copies. Phylogenetic analysis using the chloroplast genome data indicated that *R. thyrsoidea* was sister to the species in the family Malvaceae.

*Reevesia thyrsoidea* Lindl. is an important ornamental plant species mainly distributed in Guangdong and Guangxi areas of China and dispersed to east or southeast Asia. It is a shade-tolerant evergreen tree in Malvaceae (previously in Sterculiaceae) with a wide range of uses: its wood is hard and heavy with straight texture, which can be easily processed into furniture, plywood and boards; its bark fibre is strong and tough, which is a superior rope, gunny bag, and paper-making material; its twigs bear a profusion of cymose-corymbose inflorescences with white, delightfully fragrant flowers, which crowns the foliage and endows it with high aesthetic value; its seeds can be used for essential oil extraction. To facilitate its genetic research and contribute to its utilization and optimum conservation, in this study, the complete chloroplast genome of the *R. thyrsoidea* was assembled through genome skimming of the whole genome Illumina sequencing data and assessed its phylogenetic position within malvids.

A strain of *R. thyrsoidea* was sampled from Guangzhou Institute of Forestry and Landscape Architecture (113.347°E, 23.236°N), Guangzhou, China and deposited in Sun Yat-sen University Herbarium (specimen code SYS-Bore-2018-08-19). Genomic DNA was extracted from mature leaves using a modified CTAB method (Doyle and Doyle 1987), then purified to construct a DNA library before high-throughput sequencing with paired-end 150 bp on an Illumina Hiseq X10 platform (Illumina, San Diego, CA). We finally got 3.78 Gb clean data with 93.20% ≥ Q30, which was used to launch an assembly of complete chloroplast genome together with *rbc*L gene sequence of *R. thyrsoidea* (GenBank accession No. HQ415190.1) as a seed on a Perl script, NOVOPlasty (Dierckxsens et al. 2017). Online software DOGMA (Wyman et al. 2004) was used to automatically annotate the chloroplast genome, followed by manual double-check and adjustment.

The complete chloroplast genome of *R. thyrsoidea* (GenBank accession No. MH939148) was 161,786 bp in size, which comprised of one large single-copy and one small single-copy regions of 90,565 bp and 20,289 bp, respectively, separated by a pair of inverted repeat regions of 25,466 bp each. It encoded a total of 131 genes (86 protein-coding, 37 tRNA, and 8 rRNA), and 17 of them were double copies. Intron-exon structure analysis indicated that 18 unique genes (12 protein-coding genes and 6 tRNA genes) contained intron, in which three unique protein-coding genes (*rps*12, *clp*P, and *ycf*3) had two introns while the others had one intron.

After aligned with MAFFT v7.307 (Katoh and Standley 2013), RAxML (Stamatakis 2014) was applied with GTRGAMMA substitution model (Stamatakis 2014) to reconstruct a maximum-likelihood (ML) phylogeny of *R. thyrsoidea* together with 13 published complete chloroplast genome from three families (Malvaceae, Dipterocarpaceae, and Thymelaeaceae) in Malvales using three species in Brassicales as outgroup. The phylogenetic analysis indicated that *R. thyrsoidea* was clustered within the group that includes the species belonging to Malvaceae (Figure 1). The complete chloroplast genome sequence of *R. thyrsoidea* will provide a valuable genomic resource for its conservation and utilization, as well as the phylogenetic studies of malvids.

**Figure 1.**
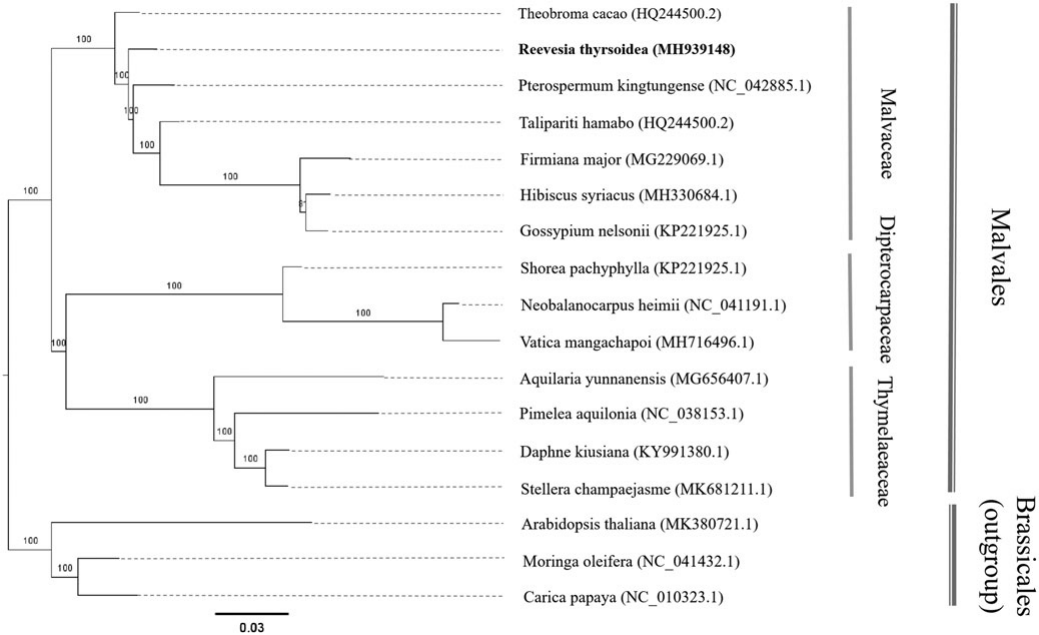
Maximum-likelihood phylogenetic tree of *R. thyrsoidea* with 13 species belonging to the Malvales based on the complete chloroplast genome sequences. Numbers in the nodes were the bootstrap values from 1000 replicates. Scale was substitutions per site.
